# Clinical Significance of Volume Status in Body Composition and Physical Performance Measurements in Hemodialysis Patients

**DOI:** 10.3389/fnut.2022.754329

**Published:** 2022-02-28

**Authors:** Jun Chul Kim, Jun Young Do, Seok Hui Kang

**Affiliations:** ^1^Division of Nephrology, Department of Internal Medicine, CHA Gumi Medical Center, CHA University, Gumi, South Korea; ^2^Division of Nephrology, Department of Internal Medicine, College of Medicine, Yeungnam University, Daegu, South Korea

**Keywords:** physical performance, hemodialysis, edema, nutrition, muscle mass

## Abstract

**Introduction:**

This study aimed to evaluate the association between volume status and body composition or physical performance measurements in hemodialysis patients.

**Methods:**

A total of 84 patients were enrolled in this study. The participants were divided into tertiles based on the edema index (extracellular water/total body water): low, middle, and high tertiles. Serum albumin and serum high-sensitivity C-reactive protein levels were measured. The appendicular lean mass index (ALM/Ht^2^, kg/m^2^) was measured using dual-energy X-ray absorptiometry. The thigh muscle area index (TMA/Ht^2^, cm^2^/m^2^) was measured using CT. Extracellular and total body water and phase angles were obtained using bioimpedance analysis. The results of the subjective global assessment (SGA), hand-grip strength (HGS), gait speed (GS), short physical performance battery (SPPB), sit-to-stand for 30-second (STS30) test, timed up and go (TUG), sit-to-stand test performed five times (STS5), and 6-minute walk (6-MW) tests were also evaluated.

**Results:**

On the univariate analysis, the SGA score and phase angle in the high tertile group were the lowest among the three groups. On multivariate analysis, TMA/Ht^2^ and phase angle in the high tertile were the lowest among the three groups. Inverse correlations were observed between edema index and TMA/Ht^2^, SGA score, phase angle, HGS, GS, SPPB, STS30, or 6-MW. Positive correlations were observed between the edema index and the STS5 or TUG test. The sensitivity and specificity for predicting low GS were 34.5 and 89.7%, respectively. The values for predicting low SPPB were 68.0 and 79.7%, respectively.

**Conclusion:**

This study demonstrates that high volume status may be associated with decreased muscle mass and physical performance regardless of inflammatory or nutritional status.

## Introduction

The number of patients undergoing hemodialysis (HD) has increased as life expectancy and the incidence of chronic diseases such as diabetes mellitus (DM) or hypertension has increased (1–3). HD patients are inherently prone to hypervolemic status due to a decrease in renal function. Hypervolemia in HD patients is associated with increased blood pressure and high cardiovascular mortality or morbidity (4, 5). Volume control in HD patients is an important issue. Recent studies have shown that hypervolemia has indirect effects on the cardiovascular system and is associated with malnutrition and/or inflammation (6).

Hypervolemia can lead to inflammation. Although the definite pathogenesis between the two variables remains unclear, some evidence has shown that the possible pathogenesis includes endotoxin translocation *via* the edematous bowel or a decrease in splanchnic blood flow by intradialytic hypotension or high ultrafiltration volume (6, 7). Consequently, inflammation can lead to malnutrition, particularly a decrease in muscle mass, which is associated with a higher prevalence of sarcopenia in patients with dialysis than in the general population (8). Some studies have reported an association between volume status and inflammation or biochemical nutritional markers. However, biochemical markers are not accurate indicators of hypervolemic status. For example, serum albumin is generally a surrogate marker of nutritional status, but serum albumin is decreased by hypervolemia and/or inflammation regardless of nutritional status (9). Therefore, body composition analysis or physical performance may be a more accurate method for predicting the changes in muscle mass or quality. This study aimed to evaluate the association between volume status and body composition or physical performance.

## Methods

### Study Population

This was a retrospective and cross-sectional study based on the analysis of an existing dataset (10). Briefly, this study was performed in a tertiary medical center between September 2012 and March 2015. We identified all adult patients undergoing HD, patients who had dialysis vintage of ≥6 months, patients who were able to ambulate without an assistive device, and patients who were not hospitalized within 3 months before enrollment. A total of 84 patients were enrolled in this study. Informed consent was obtained from all participants. This study was approved by the institutional review board of a tertiary medical center. All the investigations were conducted according to the principles of the Declaration of Helsinki. None of the participants were taking opioids, antihistamines, or antidepressants, which can be associated with decreased physical performance and cognitive function. The participants were divided into tertiles based on the edema index: low, middle, and high tertiles.

### Study Variables

Patients' demographic data were obtained: sex, age, underlying diseases of the end-stage renal disease, and dialysis duration. The following laboratory data were collected: hemoglobin (g/dl), albumin (g/dl), high-sensitivity C-reactive protein (hs-CRP) (mg/dl), blood urea nitrogen (BUN) (mg/dl), creatinine (mg/dl), aspartate transaminase (AST) (U/L), alanine transaminase (ALT) (U/L), calcium (mg/dl), phosphorus (mg/dl), sodium (mEq/l), potassium (mEq/l), chloride (mEq/l), intact parathyroid hormone (i-PTH) (pg/ml), total cholesterol (mg/dl), and spKt/V_urea_.

Serum albumin and hs-CRP levels were measured using an Olympus AU4500 automatic chemical analyzer (Olympus, Tokyo, Japan). Serum albumin level was measured using the bromocresol green method. Serum albumin and hs-CRP levels were averaged over three measurements. spKt/V_urea_ was calculated using the Daugirdas' formula (11).

### Assessment of Body Composition Measurements and Subjective Global Assessment

In this study, muscle mass was measured using whole-body dual-energy X-ray absorptiometry (DEXA) (GE Medical Systems Lunar, Madison, Wisconsin, USA) and CT (Aquilion ONE; Toshiba Medical Systems Corporation, Tokyo, Japan) of the thigh. Two measurements were performed while the patient was in the supine position, wearing a light gown midweek following the HD session. Appendicular lean mass index (ALM/Ht^2^, kg/m^2^), total fat mass index (FM/Ht^2^, kg/m^2^), and T-score were measured using DEXA. ALM/Ht^2^ and FM/Ht^2^ were calculated as the sum of the muscle mass of both extremities or the total fat mass per height squared. The bone mineral density of the whole body was measured, and the *T*-score was defined as the bone mineral density of the whole body using the International Society for Clinical Densitometry guidelines (12).

After the DEXA measurements, the patient underwent a mid-thigh CT scan. With regard to CT, axial images were obtained at the midpoint of a line extending from the superior border of the patella to the greater trochanter (3 mm thickness, 5 slices). The images were analyzed using image analysis software (ImageJ 1.45S, National Institutes of Health, Bethesda, Maryland, USA). The values were divided by height squared, and the thigh muscle area index (TMA/Ht^2^, cm^2^/m^2^) was calculated. In addition, the intermuscular fat area (IMFA) index was obtained from the same image.

The edema index, visceral fat area (VFA) (cm^2^), and phase angle were measured using multifrequency bioimpedance analysis (BIA) (InBody, Seoul, Korea). Extracellular and total body water was obtained using BIA. The edema index was defined as the ratio of extracellular water to total body water. Briefly, the BIA machine presented an edema index, and the abnormal cut-off value was obtained from the mean and SD of a healthy population with large sample size. The normal edema index was defined as the mean ± 2SD (interval of mean ± 2 SD, 0.300–0.350). Patients with an edema index > 0.350 were classified as having hypervolemia. However, the edema index is a continuous variable that should be considered for some biased measurements on a single measurement. Therefore, categorization by tertiles or quartiles rather than dichromatic approaches using a single cut-off value would be helpful in identifying trends according to a continuous variable. Therefore, we divided the patients into tertiles based on limited sample size. Levels of the edema index in low, middle, or high tertile were 0.338 ± 0.010 (interval, 0.318–0.351), 0.356 ± 0.003 (interval, 0.352–0.361), and 0.370 ± 0.007 (interval, 0.363–0.388), respectively.

Body mass index (BMI) (kg/m^2^) was calculated using body weight per height squared. Subjective global assessment (SGA) is a scoring system consisting of seven items: weight loss, dietary intake, gastrointestinal symptoms, functional capacity, comorbidity, decreased fat, or muscle (13). The 7-point SGA scale was used and defined; patients with 6–7 points were defined as well-nourished (14). Therefore, patients with an SGA score <6 points were categorized into the malnourished SGA group.

### Assessment of Handgrip Strength and Physical Performance

Hand-grip strength (HGS) was measured using a manual hydraulic dynamometer (Jamar hydraulic hand dynamometer; Sammons Preston, Chicago, Illinois, USA). Each participant performed three trials on the dominant hand and the maximum strength was recorded. Gait speed (GS) was measured according to a standard protocol; the participants were asked to walk a 4-m distance and the time taken (in seconds) to walk the distance was recorded. The short physical performance battery (SPPB) test consisted of GS, a sit-to-stand test performed five times (STS5), and balance tests and was calculated using the previously defined methods (scores ranged between 0 and 12) (15). STS5 was measured according to a previous protocol; each participant was seated on a chair with arms crossed and hands touching the shoulders (16). The participants were asked to stand up and sit down five times as quickly as possible, and the time it took for the participants to perform the tasks was recorded (in seconds).

For the sit-to-stand for 30-s (STS30), the participants were seated on a chair with their arms crossed and hands touching the shoulders. Scores were defined as the number of stands a person could complete in 30 s without using their arms to stand (17). For the 6-min walk test (6-MWT), the participants were asked to walk at their usual pace for 6 min and the distance they covered was recorded in meters (18). For the timed up and go (TUG) test, the participants were instructed to rise from an armchair, walk 3 m, turn around, return, and sit down (19). The time it took for the participants to perform the tasks was recorded (in seconds). The average step was measured using a pedometer. Moreover, a low group was defined based on their scores on the SPPB and GS tests. The low SPPB group was defined as participants with a score of ≤10, while the low GS group was defined as those with a speed of ≤0.8 m/s (20).

The presence of frailty was defined using the Johansen method (21). Briefly, slowness, poor endurance, physical inactivity, and unintentional weight loss are components of frailty. The presence of each frailty component was scored as 1, and the individual scores for each of the four components were summed. Participants with ≥3 points were defined as having frailty. Disability was measured by asking four questions related to the activities of daily living to determine whether the patient required assistance when feeding, dressing/undressing, getting in/out of bed, or taking a bath/shower (22). Each question required one of the following three responses: no help, some help, or full help. Disability was defined as requiring some or full assistance when performing two or more activities in daily living domains. In addition, we determined whether the patient had limitations in vigorous or moderate physical activity. Vigorous or moderate physical activity was defined based on the WHO guidelines (23). The participant selected one of the following three answers: severe limitation, some limitation, or no limitation.

### Statistical Analysis

Data were analyzed using the statistical software SPSS version 25 (Chicago, Illinois, USA). Categorical variables were expressed as counts (percentages) and were analyzed using the chi-square test. Continuous variables were expressed as mean ± SD or SE. For continuous variables, means were compared using one-way ANOVA, followed by a *post-hoc* Tukey comparison, and analysis of covariance for multivariate analysis. The correlation between two continuous variables was assessed using Pearson's and partial correlation analyses. The patient survival analysis was compared using the Cox regression analysis. The multivariate analysis was adjusted for age, sex, and DM. The level of statistical significance was set at *P* < 0.05.

## Results

### Participants' Clinical Characteristics

The mean age of the low tertile group was lower than that of the other tertile groups (Table 1). The proportion of DM as an underlying disease increased as the edema index tertile increased; the proportion of chronic glomerulonephritis was the greatest in the low tertile (Supplementary Table 1). The distribution of underlying diseases of end-stage renal disease was significantly different among the tertiles (*P* = 0.019). The number of male participants was similar among the three groups. There were no significant differences in hemoglobin, hs-CRP, BUN, creatinine, AST, ALT, serum calcium, phosphorus, sodium, potassium, chloride, i-PTH, total cholesterol, and spKt/V_urea_ levels among the three groups.

**Table 1:** Participants' clinical characteristics.

	**Low tertile (*n* = 26)**	**Middle tertile (*n* = 29)**	**High tertile (*n* = 29)**	***P*-value**
Sex (male, %)	13 (50.0%)	14 (48.3%)	17 (58.6%)	0.702
Age (years)	50.5 ± 12.7	58.5 ± 9.7^a^	59.9 ± 11.6^a^	0.006
DM as underlying disease of ESRD (%)	7 (26.9%)	16 (55.2%)	21 (72.4%)	0.003
Dialysis vintage (years)	4.7 ± 5.3	3.9 ± 5.0	5.0 ± 5.3	0.720
Hemoglobin (mg/dL)	11.1 ± 0.6	10.9 ± 0.6	10.9 ± 0.6	0.398
hs-CRP (mg/dL)	0.16 ± 0.10	0.18 ± 0.15	0.21 ± 0.15	0.610
Blood urea nitrogen (mg/dL)	63.3 ± 16.5	55.9 ± 16.5	55.9 ± 16.5	0.168
Creatinine (mg/dL)	11.1 ± 3.3	10.0 ± 1.8	9.9 ± 2.5	0.177
Serum albumin (g/dL)	3.8 ± 0.3	3.9 ± 0.2	3.8 ± 0.3	0.662
Aspartate transaminase (U/L)	16.0 ± 3.6	19.3 ± 6.1	18.1 ± 6.9	0.104
Alanine transaminase (U/L)	14.7 ± 7.2	15.2 ± 6.3	17.5 ± 8.8	0.335
Serum calcium (mg/dL)	8.3 ± 0.8	8.3 ± 0.5	8.5 ± 0.7	0.501
Serum phosphorus (mg/dL)	6.7 ± 0.4	6.6 ± 0.4	6.6 ± 0.4	0.442
Serum sodium (mEq/L)	137.9 ± 2.6	136.9 ± 2.8	138.1 ± 2.8	0.223
Serum potassium (mEq/L)	4.9 ± 0.5	5.1 ± 0.4	5.0 ± 0.7	0.403
Serum chloride (mEq/L)	98.8 ± 3.4	98.0 ± 3.2	98.7 ± 3.7	0.645
Intact parathyroid hormone (pg/mL)	281.5 ± 198.3	240.4 ± 144.0	270.6 ± 207.4	0.691
Total cholesterol (mg/dL)	162.2 ± 32.4	151.1 ± 34.1	149.2 ± 36.1	0.326
spKt/V_urea_	1.43 ± 0.3	1.35 ± 0.4	1.32 ± 0.2	0.417

*Data are expressed as mean ± SD for continuous variables and numbers (percentage) for categorical variables. P-values were tested by one-way ANOVA, followed by a post-hoc Tukey comparison for continuous variables, and Pearson's χ^2^ or Fisher's exact tests for categorical variables*.

a *P < 0.05 compared with low tertile*.

*DM, diabetes mellitus; ESRD, end-stage renal disease; hs-CRP, high-sensitivity C-reactive protein*.

### Association Between Edema Index and Body Composition, Nutritional, or Physical Performance Indices

On the univariate analysis, the SGA score and phase angle in the high tertile were the lowest among the three groups (Table 2). On multivariate analysis, TMA/Ht^2^ and phase angle in the high tertile were the lowest among the three groups. There were no significant differences in BMI, ALM/Ht^2^, FM/Ht^2^, VFA, T-score of BMD, and serum albumin levels among the three groups. The IMFA index was greatest in the high tertile among tertiles in univariate and multivariate analyses.

**Table 2:** Comparison of body composition and nutritional indices according to edema index tertile.

	**Low tertile**	**Middle tertile**	**High tertile**	***P*-value**
**Univariate**				
Body mass index (kg/m^2^)	23.3 ± 3.0	23.5 ± 3.4	24.3 ± 4.3	0.538
ALM/Ht^2^ (kg/m^2^)	6.7 ± 1.1	6.5 ± 0.9	6.6 ± 0.9	0.685
FM/Ht^2^ (kg/m^2^)	6.7 ± 2.9	6.7 ± 2.8	6.8 ± 3.5	0.995
TMA/Ht^2^ (cm^2^/m^2^)	39.1 ± 8.1	37.2 ± 6.4	34.6 ± 6.1	0.052
Visceral fat area (cm^2^)	88.0 ± 23.6	96.8 ± 25.8	104.3 ± 30.6	0.087
*T*-score of BMD	−0.9 ± 1.7	−0.9 ± 1.4	−1.1 ± 1.4	0.845
SGA score	6.1 ± 1.0	5.7 ± 1.0	5.3 ± 0.9^a^	0.009
Serum albumin (g/dL)	3.8 ± 0.3	3.9 ± 0.2	3.8 ± 0.3	0.662
Phase angle	5.9 ± 0.9	4.7 ± 0.5^a^	4.2 ± 0.8^a^^,^^b^	<0.001
IMFA index (cm/m^2^)	1.6 ± 0.8	1.7 ± 1.0	2.6 ± 2.0^a^^,^^b^	0.015
**Multivariate**				
Body mass index (kg/m^2^)	23.5 ± 0.8	23.5 ± 0.7	24.1 ± 0.7	0.797
ALM/Ht^2^ (kg/m^2^)	6.6 ± 0.2	6.5 ± 0.1	6.6 ± 0.2	0.873
FM/Ht^2^ (kg/m^2^)	6.7 ± 0.6	6.6 ± 0.5	6.8 ± 0.6	0.943
TMA/Ht^2^ (cm^2^/m^2^)	39.1 ± 1.4	37.5 ± 1.2	34.3 ± 1.2^a^^,^^b^	0.041
Visceral fat area (cm^2^)	94.8 ± 5.2	96.0 ± 4.6	99.1 ± 4.8	0.827
*T*-score of BMD	−1.0 ± 0.3	−0.8 ± 0.3	−1.0 ± 0.3	0.786
SGA score	6.0 ± 0.2	5.8 ± 0.2	5.4 ± 0.2	0.093
Serum albumin (g/dL)	3.8 ± 0.1	3.9 ± 0.0	3.9 ± 0.1	0.266
Phase angle	6.0 ± 0.1	4.7 ± 0.1^a^	4.1 ± 0.1^a^^,^^b^	<0.001
IMFA index (cm/m^2^)	1.7 ± 0.3	1.6 ± 0.3	2.6 ± 0.3^a^^,^^b^	0.037

*Data were expressed as the mean ± SD for univariate analysis and the mean ± SE for multivariate analysis. Univariate analysis was performed using one-way ANOVA, followed by a post-hoc Tukey comparison, and multivariate analysis was performed using analysis of covariance and adjusted for age, sex, and diabetes mellitus*.

a *P < 0.05 compared with low tertile*.

b *P < 0.05 compared with middle tertile*.

*ALM/Ht^2^, appendicular lean mass per height squared; FM/Ht^2^, fat mass per height squared; TMA/Ht^2^, thigh muscle area per height squared; BMD, bone mineral density; SGA, subjective global assessment; IMFA, intermuscular fat area*.

HGS, GS, SPPB, STS30, and 6-MWT in the high tertile were the lowest among the three groups in the univariate and multivariate analyses (Table 3). There was no significant difference in the average number of steps among the three groups. TUG and STS5 in the high tertile were the greatest among the three groups in the univariate analysis but not in the multivariate analysis. The phase angle values in the low, middle, and high tertiles were 5.9 ± 0.9, 4.7 ± 0.2, and 4.2 ± 0.8, respectively (*P* < 0.001). The multivariate analyses showed trends similar to those from the univariate analysis.

**Table 3:** Comparison of physical performance indices according to edema index tertile.

	**Low tertile**	**Middle tertile**	**High tertile**	***P*-value**
**Univariate**				
HGS	29.4 ± 9.3	26.0 ± 5.5	23.1 ± 5.8^a^	0.005
Gait speed	0.99 ± 0.17	0.96 ± 0.15	0.81 ± 0.22^a^^,^^b^	0.001
SPPB	11.6 ± 0.9	11.4 ± 0.9	9.7 ± 2.1^a^^,^^b^	<0.001
STS5	7.2 ± 2.1	8.2 ± 2.4	11.1 ± 9.7^a^	0.046
STS30	20.7 ± 6.0	17.9 ± 4.7	15.2 ± 5.2^a^	0.001
6-MWT	505.4 ± 87.7	482.2 ± 92.7	394.1 ± 124.6^a^^,^^b^	<0.001
TUG	6.3 ± 1.6	7.1 ± 1.6	8.5 ± 2.2^a^^,^^b^	<0.001
Average steps	5,882 ± 2,659	5,073 ± 3,509	3,862 ± 3,815	0.097
**Multivariate**				
HGS	28.6 ± 1.2	26.6 ± 1.0	23.2 ± 1.1^a^^,^^b^	0.005
Gait speed	0.96 ± 0.04	0.97 ± 0.03	0.82 ± 0.03^a^^,^^b^	0.002
SPPB	11.3 ± 0.3	11.5 ± 0.3	9.9 ± 0.3^a^^,^^b^	<0.001
STS5	8.0 ± 1.3	8.0 ± 1.1	10.5 ± 1.2	0.227
STS30	20.0 ± 1.1	18.2 ± 1.0	15.5 ± 1.0^a^^,^^b^	0.018
6-MWT	478.0 ± 20.0	491.7 ± 17.5	409.1 ± 18.2^a^^,^^b^	0.004
TUG	6.8 ± 0.4	6.9 ± 0.3	8.3 ± 0.3^a^^,^^b^	0.005
Average steps	5,939 ± 757	5,040 ± 646	3,845 ± 683	0.149

*Data are expressed as mean ± SD for univariate analysis and the mean ± SE for multivariate analysis. Univariate analysis was performed using one-way ANOVA, followed by a post-hoc Tukey comparison, and multivariate analysis was performed using analysis of covariance and adjusted for age, sex, and diabetes mellitus*.

a *P < 0.05 compared with low tertile*.

b *P < 0.05 compared with middle tertile*.

*HGS, handgrip strength; SPPB, short physical performance battery; STS5, five times sit-to-stand test; STS30, sit-to-stand for 30-s test; 6-MWT, 6-min walk test; TUG, timed up-to-go test*.

The correlations between the edema index and various indices are shown in Supplementary Table 2. Inverse correlations were observed between the edema index and TMA/Ht^2^, SGA score, phase angle, HGS, GS, SPPB, STS30, or 6-MWT. Positive correlations were observed between the edema index and the STS5 or TUG test.

The number of patients with disability in the low, middle, and high tertiles was 1 (3.8%), 1 (3.4%), and 3 (10.3%), respectively (*P* = 0.465). The prevalence of frailty in the low, middle, and high tertiles was 3 (11.5%), 7 (24.1%), and 14 (48.3%), respectively (*P* = 0.009). Those with the low GS in low, middle, or high tertiles were 4 (15.4%), 6 (20.7%), and 17 (58.6%), respectively (*P* = 0.001). Those with low SPPB in the low, middle, or high tertiles were 2 (7.7%), 6 (20.7%), and 17 (58.6%), respectively (*P* < 0.001).

The discrimination power of the edema index for predicting disability, frailty, low GS, or low SPPB was determined using receiver operating characteristic (ROC) analysis. The area under the ROC, sensitivity, and specificity for predicting disability were 0.691 (0.581–0.787), 50 (14.7–94.7)%, and 79.9 (69.2–88.0)%, respectively (*P* = 0.183). Those for predicting frailty were 0.713 (0.604–0.806), 66.7 (44.7–84.4)%, and 71.7 (58.6–82.5)%, respectively (*P* < 0.001). Those for predicting low GS were 0.641 (0.529–0.743), 34.5 (22.2–48.6)%, and 89.7 (72.6–97.8)%, respectively (*P* = 0.025). Those for predicting low SPPB were 0.791 (0.688–0.872), 68.0 (46.5–85.1)%, and 79.7 (67.2–89.0)%, respectively (*P* < 0.001).

Supplementary Table 3 shows the questionnaire results of the subjective evaluation of the limitations in physical activity. High tertile was associated with a higher limitation of physical activity compared with the other tertiles. In addition, in univariate analysis, the odds ratio of high tertile of the edema index was 2.76 (95% CI, 0.97–7.84; *P* = 0.058) for low GS, 8.32 (95% CI, 2.91–23.85; *P* < 0.001) for low SPPB, 4.20 (95% CI, 1.55–11.42; *P* = 0.005) for frailty, 3.06 (95% CI, 0.48–19.44; *P* = 0.236) for disability, and 2.33 (95% CI, 0.95–5.85; *P* = 0.071) for malnourished SGA. In multivariate analyses, the odds ratio of high tertile of the edema index was 3.29 (95% CI, 0.98–11.02; *P* = 0.053) for low GS, 6.30 (95% CI, 2.08–19.11; *P* = 0.001) for low SPPB, 3.00 (95% CI, 1.01–8.88; *P* = 0.047) for frailty, 1.26 (95% CI, 0.16–10.23; *P* = 0.828) for disability, and 1.83 (95% CI, 0.67–4.98; *P* = 0.237) for malnourished SGA.

### Subgroup Analyses by DM and Age

For participants without DM, multivariate analyses showed inverse correlations between the edema index and TMA/Ht^2^, SGA score, phase angle, HGS, GS, SPPB, or STS30 (Supplementary Table 4). Positive correlations were observed between the edema index and the STS5 or TUG test. For participants with DM, multivariate analyses showed inverse correlations between the edema index and phase angle, SPPB, STS30, or 6-MWT. Positive correlations were observed between the edema index and the STS5 or TUG test.

For participants aged < 65 years, the multivariate analyses showed inverse correlations between edema index and TMA/Ht^2^, SGA score, phase angle, HGS, GS, SPPB, STS30, or 6-MWT (Supplementary Table 5). Positive correlations were observed between the edema index and the STS5 or TUG test. For participants aged ≥65 years, multivariate analyses showed inverse correlations between the edema index and SGA score, phase angle, GS, or average steps.

For participants <3 years of dialysis vintage (*n* = 42), the multivariate analyses showed inverse correlations between the edema index and phase angle, HGS, SPPB, STS30, or average steps (Supplementary Table 6). For participants ≥3 years of dialysis vintage (*n* = 42), the multivariate analyses showed inverse correlations between the edema index and TMA/Ht^2^, SGA score, phase angle, GS, SPPB, STS30, or 6-MWT.

### Survival Analyses According to Variables

The mean follow-up durations in the low, middle, and high tertiles were 646 ± 317, 650 ± 321, and 491 ± 356 days (*P* = 0.128). Survival analyses were performed using Cox regression (Supplementary Table 7). The univariate Cox regression analyses showed that an increase in 0.01 unit of edema index was associated with a 2.19 higher hazard ratio and the multivariate analysis showed a 2.26 higher hazard ratio according to an increase in 0.01 unit of edema index. On the univariate analysis, increases in GS, STS30, and 6-MWT were associated with a lower hazard ratio. The multivariate analyses showed trends similar to those from the univariate analyses.

## Discussion

In this study, the participants were divided into three tertiles according to the edema index. Baseline age and the presence of diabetes differed among the three groups. Multivariate analyses adjusted for age, sex, and presence of DM were performed. The results of multivariate analyses showed that the edema index was associated with TMA/Ht^2^, phase angle, and most physical performance measurements but not with BMI, FM/Ht^2^, ALM/Ht^2^, VFA, T-score of BMD, SGA score, hs-CRP, and serum albumin level. In addition, correlation analyses were performed using the edema index as a continuous variable and subgroup analyses for the presence of DM or age. Results from the correlation and subgroup analyses showed similar trends. The discrimination power of the edema index for predicting frailty, low GS, or low SPPB was relatively favorable. Furthermore, this study showed an association between edema index and mortality.

First, we evaluated the association between edema index and body composition measurements. There was no significant difference in visceral fat, total fat mass index, or bone components among the three groups. Muscle mass measurement among the three groups was different in TMA/Ht^2^ alone using CT and not in ALM/Ht^2^ using DEXA. Using DEXA, lean mass was measured as all masses except bone and fat. It includes the skin and fat-free mass of the adipose tissue (24). Volume overload can distribute muscle mass and skin, which leads to an overestimation of lean mass using DEXA. However, muscle mass using CT can exclude overhydrated skin and fat-free mass of adipose tissue. In this data, the non-difference in ALM/Ht^2^ using DEXA may be associated with the overestimation of measurements according to volume overload. The difference was clearer for measurements using CT than for DEXA.

Other important results of this study were FM, IMFA indices, and mortality. Fat measurements using CT or DEXA were relatively accurate, regardless of the volume effects. There was no significant difference in the FM index among tertiles, but the IMFA index was the greatest in the high tertile among tertiles. Hypervolemia is associated with inflammation, which can lead to insulin resistance and/or cardiovascular disease. Previous studies have shown that volume overload contributes to the pathogenesis of insulin resistance in HD patients, which results in a high prevalence of cardiovascular disease (6, 25, 26). This study revealed that patients with a high-volume status had a greater IMFA index than those with other tertiles. This may be associated with decreased muscle function (27). However, a difference in the FM index was not obtained among tertiles. The combination effect with an increase in fat by insulin resistance and decrease in fat by malnutrition would be associated with non-difference in FM index among tertiles.

In addition, we compared the associations between various variables and mortality rates. An increase in the edema index was associated with a high mortality on univariate and multivariate analyses. High mortality in patients with a high edema index would be associated with combined effects, including cardiovascular effects, inflammation, and/or malnutrition. An increase in GS, STS30, or 6-MWT was associated with low mortality in the univariate and multivariate analyses. These findings reveal that favorable physical performance is associated with improved patient survival. However, some indicators such as HGS, SPPB, STS5, TUG, and muscle mass indices were not statistically significant. Various factors, such as small differences in variables between participants, small sample size, or overestimated muscle mass measurements due to volume overload, may lead to statistical non-significance.

Another important result in this study is the inverse association between the phase angle and volume status. Previous studies have shown an association between phase angle and prognosis, including nutritional status and mortality in dialysis patients (28, 29). The phase angle is originally associated with cellularity and water content to cell mass, and a high phase angle reveals high cellularity and low extracellular water to intracellular water ratio (30). Therefore, the phase angle can be influenced by the nutritional and volume status in dialysis patients. The slope of extracellular water to body weight in patients with dialysis increases more steeply compared with normovolemia participants (31). These results reveal that the edema index increased as the volume overloading increased, which resulted in a decrease in the phase angle. In addition, patients with hypervolemic can be associated with malnutrition, which leads to cellular degradation, which, in turn, results in a low phase angle through low cellularity. Our results showed that the phase angle decreased as the edema index tertile increased, which was similar to the results of previous studies.

This study showed that the association between the edema index and SGA was only modest, and there was no association between the edema index and serum albumin or hs-CRP. Previous studies have shown a positive association between volume overload and inflammation or hypoalbuminemia in HD patients (26, 32, 33). The association between volume status and serum albumin level was bidirectional. Volume overloading can lead to underestimation of the actual serum albumin level, and hypoalbuminemia can be associated with increased extracellular water *via* decreased osmotic pressure. The inverse association between the two variables would be reasonable. However, this study did not show a strong association among these variables, which may be due to the relatively stable volume status in our cohort and the measurement of two variables in the post-HD period. As the volume overload increased, the effect on serum albumin of volume increased. We measured the edema index post-HD, and the edema index interval in our cohort was 0.318–0.388 (normal range proposed from BIA: 0.300–0.350). Therefore, the post-HD edema index in one-third of our cohort was within the normal range. In the post-HD period, differences between patients with the lowest and highest values would not be sufficiently large to induce statistical significance. However, the trends for hs-CRP were similar to those for the edema index. If these variables were measured during the pre-HD period, statistical significance may have been obtained.

This study showed a negative association between the edema index and nutritional or inflammatory indices, but physical performance indices and muscle mass decreased as the edema index increased. These findings reveal that the occurrence of non-severe overhydration in our cohort did not lead to biochemical changes, but this can lead to a decrease in physical performance, which is associated with a decrease in muscle mass or function. Subjective sensation for limitation of physical activity was also greater in the high tertile group than in the other tertile groups.

In this study, the number of participants differed among the three groups. The edema index was calculated as extracellular water/total body water, and the same absolute value was difficult to obtain. However, the BIA machine expresses the edema index to only three decimal points in error or validity to calculate the edema index. Some patients had the same edema index values, despite having different absolute values. Considering the limitations of categorical groups, we performed additional analyses for the association between edema index values as continuous variables and various indices.

The strength of this study is its comprehensive evaluation of physical performance. Physical performance is inversely associated with muscle strength and/or mass (34, 35). However, a decrease in muscle strength occurs earlier than a decrease in muscle mass. Decreased physical performance is correlated with decreased muscle strength, but the association is non-linear. Physical performance is more likely to be associated with muscle quality than muscle mass *per se*. In addition, physical performance would be generally influenced by various factors, such as lifestyle and biological and psychological factors. Beyond muscle mass or strength, abnormalities in motor coordination, excitation-contraction coupling, and skeletal integrity can lead to a decrease in physical performance. Therefore, a decrease in physical performance can develop before or without a decrease in muscle mass or strength. In this study, the strong positive association between the edema index and strength or physical performance indices, and the weak positive association between the edema index and muscle mass index may be parallel to these concepts.

This study has some inherent limitations. First, this study was performed at a single center and had a small sample size. This study was a cross-sectional retrospective study using a cohort from a previous study (10). This design does not evaluate the causal relationship among variables. In addition, the small sample size could limit the adjustment of confounding variables. However, there were significant differences in baseline age and the presence of DM among the tertiles in this study. Differences in two variables can lead to a statistical bias, and sex can also be considered as a confounding factor for the comparison of body composition, nutrition, and physical performance indicators. The results may not be accurate, without adjustment for these variables. Statistical significance was obtained despite the small sample size. Second, the volume status in this study was measured post-HD. Measurements including volume status, muscle mass, strength, and physical performance were evaluated at midweek following the HD session. Single measurements may not completely reflect the actual volume status. In addition, this study did not evaluate the association between longitudinal changes in volume status, such as before and after HD session, or changes in volume status after HD session, and outcomes. The application of time-averaged volume status using measurements at some points between HD sessions or analyses using longitudinal data would be useful in overcoming these limitations. A large longitudinal study, including large sample size and both pre- and post-HD measurements, or longitudinal changes in volume status, is warranted to overcome these limitations.

In conclusion, this study demonstrates that high volume status may be associated with a decrease in muscle mass and physical performance regardless of inflammatory or nutritional status.

## Data Availability Statement

The original contributions presented in the study are included in the article/Supplementary Material, further inquiries can be directed to the corresponding author/s.

## Ethics Statement

This study was approved by the Institutional Review Board of CHA Gumi Medical Center. The patients/participants provided their written informed consent to participate in this study.

## Author Contributions

SK: research idea, study design, and data analysis and interpretation. JK: data acquisition, supervision or mentorship, and takes responsibility that this study has been reported honestly, accurately, transparently, and accepts accountability for the overall work by ensuring that questions pertaining to the accuracy or integrity of any portion of the work are appropriately investigated and resolved. JD: statistical analysis. SK and JD: wrote and revised the manuscript. All authors contributed to the article and approved the submitted version.

## Funding

This study was supported by the Medical Research Center Program (2015R1A5A2009124) through the National Research Foundation of Korea (NRF) funded by the Ministry of Science, ICT and Future Planning. The funder had no role in the study design, data collection, analysis, interpretation, manuscript writing, or decision to submit the article for publication.

## Conflict of Interest

The authors declare that the research was conducted in the absence of any commercial or financial relationships that could be construed as a potential conflict of interest.

## Publisher's Note

All claims expressed in this article are solely those of the authors and do not necessarily represent those of their affiliated organizations, or those of the publisher, the editors and the reviewers. Any product that may be evaluated in this article, or claim that may be made by its manufacturer, is not guaranteed or endorsed by the publisher.
