# Supplementary material for: Clinical Significance of Volume Status in Body Composition and Physical Performance Measurements in Hemodialysis Patients
Source: Front Nutr. 2022 Feb 28;9:754329. doi: 10.3389/fnut.2022.754329 (PMC8922218; doi:10.3389/fnut.2022.754329)
Supplement: Supplementary file 1 [file Table_1.docx]

**Supplementary Table 1. Underlying diseases of the end-stage renal disease**

|  | **Low tertile** | **Middle tertile** | **High tertile** | ***P*-value** |
| --- | --- | --- | --- | --- |
| DM | 7 (26.9%) | 16 (55.2%) | 21 (72.4%) | 0.019 |
| CGN | 11 (42.3%) | 10 (34.5%) | 0 (%) |  |
| HTN | 2 (7.7%) | 2 (6.9%) | 1 (3.4%) |  |
| ADPKD | 1 (3.8%) | 1 (3.4%) | 2 (6.9%) |  |
| Gout | 1 (3.8%) | 0 (%) | 2 (6.9%) |  |
| Ischemic RD | 0 (%) | 0 (%) | 1 (3.4%) |  |
| Renal TB | 1 (3.8%) | 0 (%) | 0 (%) |  |
| Unknown | 3 (11.5%) | 0 (%) | 2 (6.9%) |  |

Abbreviations: DM, diabetes mellitus; CGN, chronic glomerulonephritis; HTN, hypertension; ADPKD, autosomal dominant polycystic kidney disease; Ischemic RD, ischemic renal disease; TB, tuberculosis.

**Supplementary Table 2. Correlation between edema index and various indices**

|  | **Univariate** | |  | **Multivariate** | |
| --- | --- | --- | --- | --- | --- |
|  | ***r*** | ***P*-value** |  | ***r*** | ***P*-value** |
| Body mass index | 0.015 | 0.891 |  | –0.048 | 0.676 |
| ALM/Ht^2^ | –0.037 | 0.740 |  | –0.026 | 0.819 |
| FM/Ht^2^ | –0.097 | 0.380 |  | –0.123 | 0.284 |
| TMA/Ht^2^ | –0.332 | 0.002 |  | –0.444 | <0.001 |
| Visceral fat area | 0.144 | 0.191 |  | –0.021 | 0.852 |
| T-score of BMD | –0.032 | 0.770 |  | 0.021 | 0.857 |
| SGA score | –0.441 | <0.001 |  | –0.358 | 0.001 |
| Serum albumin | 0.022 | 0.842 |  | 0.079 | 0.490 |
| Phase angle | –0.807 | <0.001 |  | –0.868 | <0.001 |
| Handgrip strength | –0.362 | 0.001 |  | –0.320 | 0.004 |
| Gait speed | –0.449 | <0.001 |  | –0.383 | 0.001 |
| SPPB | –0.444 | <0.001 |  | –0.333 | 0.003 |
| STS5 | 0.328 | 0.002 |  | 0.256 | 0.024 |
| STS30 | –0.493 | <0.001 |  | –0.444 | <0.001 |
| 6-MWT | –0.401 | <0.001 |  | –0.271 | 0.016 |
| TUG | 0.421 | <0.001 |  | 0.290 | 0.010 |
| Average steps | –0.188 | 0.091 |  | –0.167 | 0.144 |
| hs-CRP | 0.107 | 0.398 |  | 0.037 | 0.744 |

Correlation analyses were performed using Pearson’s correlation on univariate and partial correlations on multivariate analysis. Multivariate analysis was adjusted for age, sex, and the presence of diabetes mellitus.

Abbreviations: ALM/Ht^2^, appendicular lean mass per height squared; FM/Ht^2^, fat mass per height squared; TMA/Ht^2^, thigh muscle area per height squared; BMD, bone mineral density; SGA, subjective global assessment; SPPB, short physical performance battery; STS5, five times sit-to-stand test; STS30, sit-to-stand for 30-second test; 6-MWT, 6-minute walk test; TUG, timed up-to-go test; hs-CRP, high-sensitivity C-reactive protein.

**Supplementary Table 3. Limitations on physical activity according to edema index tertile**

|  | **Low tertile** | **Middle tertile** | **High tertile** | ***P*-value** |
| --- | --- | --- | --- | --- |
| **Limitation of vigorous physical activity** |  |  |  | 0.210 |
| Severe limitation | 12 (46.2%) | 11 (37.9%) | 19 (65.5%) |  |
| Some limitation | 9 (34.6%) | 14 (48.3%) | 6 (20.7%) |  |
| No limitation | 5 (19.2%) | 6 (20.7%) | 4 (13.8%) |  |
| **Limitation of moderate physical activity** |  |  |  | 0.018 |
| Severe limitation | 0 | 1 (3.4%) | 5 (17.2%) |  |
| Some limitation | 1 (3.8%) | 6 (20.7%) | 6 (20.7%) |  |
| No limitation | 25 (96.2%) | 22 (75.9%) | 18 (62.1%) |  |

**Supplementary Table 4. Correlation between edema index and various indices by the presence of DM**

|  | **Participants without DM** | | | |  | **Participants with DM** | | | |
| --- | --- | --- | --- | --- | --- | --- | --- | --- | --- |
|  | **Univariate** | | **Multivariate** | |  | **Univariate** | | **Multivariate** | |
|  | ***r*** | ***P*-value** | ***r*** | ***P*-value** |  | ***r*** | ***P*-value** | ***r*** | ***P*-value** |
| Body mass index | –0.310 | 0.051 | –0.227 | 0.228 |  | 0.166 | 0.282 | 0.089 | 0.647 |
| ALM/Ht^2^ | –0.325 | 0.041 | –0.305 | 0.102 |  | 0.242 | 0.114 | 0.084 | 0.666 |
| FM/Ht^2^ | –0.199 | 0.219 | –0.163 | 0.391 |  | –0.013 | 0.936 | –0.002 | 0.993 |
| TMA/Ht^2^ | –0.687 | <0.001 | –0.677 | <0.001 |  | 0.057 | 0.711 | –0.126 | 0.516 |
| Visceral fat area | –0.097 | 0.553 | –0.189 | 0.318 |  | 0.234 | 0.127 | 0.112 | 0.564 |
| T-score of BMD | –0.305 | 0.056 | –0.187 | 0.323 |  | 0.224 | 0.144 | 0.040 | 0.838 |
| SGA score | –0.658 | <0.001 | –0.506 | 0.004 |  | –0.125 | 0.419 | –0.316 | 0.095 |
| Serum albumin | –0.237 | 0.141 | –0.048 | 0.799 |  | 0.275 | 0.071 | 0.270 | 0.156 |
| Phase angle | –0.915 | <0.001 | –0.897 | <0.001 |  | –0.676 | <0.001 | –0.692 | <0.001 |
| Handgrip strength | –0.487 | 0.001 | –0.396 | 0.030 |  | –0.063 | 0.685 | –0.258 | 0.176 |
| Gait speed | –0.592 | <0.001 | –0.425 | 0.019 |  | –0.248 | 0.105 | –0.341 | 0.071 |
| SPPB | –0.520 | 0.001 | –0.385 | 0.036 |  | –0.359 | 0.017 | –0.610 | <0.001 |
| STS5 | 0.527 | <0.001 | 0.477 | 0.008 |  | 0.310 | 0.041 | 0.612 | <0.001 |
| STS30 | –0.603 | <0.001 | –0.582 | 0.001 |  | –0.320 | 0.034 | –0.517 | 0.004 |
| 6-MWT | –0.524 | 0.001 | –0.338 | 0.067 |  | –0.202 | 0.189 | –0.460 | 0.012 |
| TUG | 0.496 | 0.001 | 0.468 | 0.009 |  | 0.255 | 0.094 | 0.489 | 0.007 |
| Average steps | –0.387 | 0.014 | –0.270 | 0.149 |  | –0.026 | 0.871 | –0.068 | 0.726 |
| hs-CRP | 0.317 | 0.077 | 0.090 | 0.591 |  | –0.113 | 0.533 | –0.073 | 0.646 |

Correlation analyses were performed using Pearson’s correlation on univariate and partial correlations on multivariate analysis. Multivariate analysis was adjusted for age and sex.

Abbreviations: DM, diabetes mellitus; *r*, correlation coefficient; ALM/Ht^2^, appendicular lean mass per height squared; FM/Ht^2^, fat mass per height squared; TMA/Ht^2^, thigh muscle area per height squared; BMD, bone mineral density; SGA, subjective global assessment; SPPB, short physical performance battery; STS5, five times sit-to-stand test; STS30, sit-to-stand for 30-second test; 6-MWT, 6-minute walk test; TUG, timed up-to-go test; hs-CRP, high-sensitivity C-reactive protein.

**Supplementary Table 5. Correlation between edema index and various indices by age**

|  | **Participants < 65 years old** | | | |  | **Participants ≥ 65 years old** | | | |
| --- | --- | --- | --- | --- | --- | --- | --- | --- | --- |
|  | **Univariate** | | **Multivariate** | |  | **Univariate** | | **Multivariate** | |
|  | ***r*** | ***P*-value** | ***r*** | ***P*-value** |  | ***r*** | ***P*-value** | ***r*** | ***P*-value** |
| Body mass index | –0.053 | 0.686 | –0.143 | 0.349 |  | 0.321 | 0.136 | 0.195 | 0.503 |
| ALM/Ht^2^ | 0.036 | 0.783 | –0.187 | 0.219 |  | –0.164 | 0.454 | –0.246 | 0.396 |
| FM/Ht^2^ | –0.230 | 0.075 | –0.192 | 0.207 |  | 0.364 | 0.088 | 0.344 | 0.229 |
| TMA/Ht^2^ | –0.287 | 0.025 | –0.467 | 0.001 |  | –0.418 | 0.047 | –0.531 | 0.050 |
| Visceral fat area | 0.049 | 0.709 | –0.032 | 0.834 |  | 0.038 | 0.071 | 0.200 | 0.493 |
| T-score of BMD | –0.048 | 0.711 | –0.192 | 0.207 |  | 0.223 | 0.306 | 0.042 | 0.886 |
| SGA score | –0.415 | 0.001 | –0.405 | 0.006 |  | –0.414 | 0.050 | –0.590 | 0.026 |
| Serum albumin | 0.078 | 0.548 | –0.035 | 0.821 |  | 0.166 | 0.450 | 0.085 | 0.774 |
| Phase angle | –0.863 | <0.001 | –0.874 | <0.001 |  | –0.476 | 0.022 | –0.742 | 0.002 |
| Handgrip strength | –0.373 | 0.003 | –0.516 | <0.001 |  | –0.213 | 0.329 | 0.021 | 0.942 |
| Gait speed | –0.395 | 0.002 | –0.382 | 0.010 |  | –0.556 | 0.006 | –0.539 | 0.047 |
| SPPB | –0.415 | 0.001 | –0.522 | <0.001 |  | –0.537 | 0.008 | –0.402 | 0.154 |
| STS5 | 0.474 | <0.001 | 0.658 | <0.001 |  | 0.408 | 0.054 | 0.354 | 0.215 |
| STS30 | –0.507 | <0.001 | –0.655 | <0.001 |  | –0.371 | 0.081 | –0.420 | 0.135 |
| 6-MWT | –0.313 | 0.014 | –0.410 | 0.005 |  | –0.610 | 0.002 | –0.468 | 0.092 |
| TUG | 0.356 | 0.005 | 0.494 | 0.001 |  | 0.557 | 0.006 | 0.504 | 0.066 |
| Average steps | –0.105 | 0.422 | –0.065 | 0.672 |  | –0.508 | 0.016 | –0.548 | 0.043 |
| hs-CRP | 0.148 | 0.316 | 0.013 | 0.925 |  | –0.061 | 0.817 | 0.032 | 0.892 |

Correlation analyses were performed using Pearson’s correlation on univariate and partial correlations on multivariate analysis. Multivariate analysis was adjusted for age, sex, and the presence of diabetes mellitus.

Abbreviations: *r*, correlation coefficient; ALM/Ht^2^, appendicular lean mass per height squared; FM/Ht^2^, fat mass per height squared; TMA/Ht^2^, thigh muscle area per height squared; BMD, bone mineral density; SGA, subjective global assessment; SPPB, short physical performance battery; STS5, five times sit-to-stand test; STS30, sit-to-stand for 30-second test; 6-MWT, 6-minute walk test; TUG, timed up-to-go test; hs-CRP, high-sensitivity C-reactive protein.

**Supplementary Table 6. Correlation between edema index and various indices by the dialysis vintage**

|  | **Participants with < 3 years** | | | |  | **Participants with ≥ 3 years** | | | |
| --- | --- | --- | --- | --- | --- | --- | --- | --- | --- |
|  | **Univariate** | | **Multivariate** | |  | **Univariate** | | **Multivariate** | |
|  | ***r*** | ***P*-value** | ***r*** | ***P*-value** |  | ***r*** | ***P*-value** | ***r*** | ***P*-value** |
| Body mass index | 0.164 | 0.298 | 0.166 | 0.332 |  | –0.073 | 0.646 | –0.167 | 0.309 |
| ALM/Ht^2^ | –0.013 | 0.934 | 0.088 | 0.612 |  | –0.041 | 0.794 | –0.035 | 0.833 |
| FM/Ht^2^ | 0.081 | 0.612 | 0.122 | 0.479 |  | –0.202 | 0.200 | –0.249 | 0.127 |
| TMA/Ht^2^ | –0.119 | 0.453 | –0.072 | 0.687 |  | –0.472 | 0.002 | –0.458 | 0.003 |
| Visceral fat area | 0.415 | 0.006 | 0.262 | 0.122 |  | –0.029 | 0.959 | –0.202 | 0.218 |
| T-score of BMD | –0.092 | 0.561 | 0.023 | 0.893 |  | 0.008 | 0.959 | 0.049 | 0.768 |
| SGA score | –0.321 | 0.038 | –0.099 | 0.567 |  | –0.519 | <0.001 | –0.428 | 0.007 |
| Serum albumin | –0.295 | 0.058 | –0.254 | 0.135 |  | 0.195 | 0.217 | 0.203 | 0.216 |
| Phase angle | –0.683 | <0.001 | –0.766 | <0.001 |  | –0.864 | <0.001 | –0.896 | <0.001 |
| Handgrip strength | –0.377 | 0.014 | –0.404 | 0.015 |  | –0.357 | 0.020 | –0.270 | 0.097 |
| Gait speed | –0.250 | 0.110 | –0.063 | 0.716 |  | –0.612 | <0.001 | –0.554 | <0.001 |
| SPPB | –0.418 | 0.006 | –0.343 | 0.040 |  | –0.460 | 0.002 | –0.335 | 0.037 |
| STS5 | 0.268 | 0.086 | 0.282 | 0.096 |  | 0.358 | 0.020 | 0.259 | 0.112 |
| STS30 | –0.281 | 0.071 | –0.363 | 0.030 |  | –0.599 | <0.001 | –0.545 | <0.001 |
| 6-MWT | –0.305 | 0.049 | –0.108 | 0.529 |  | –0.456 | 0.002 | –0.326 | 0.043 |
| TUG | 0.474 | 0.002 | 0.298 | 0.077 |  | 0.394 | 0.010 | 0.280 | 0.084 |
| Average steps | –0.395 | 0.012 | –0.451 | 0.006 |  | –0.100 | 0.528 | –0.076 | 0.644 |
| hs-CRP | –0.139 | 0.379 | –0.098 | 0.571 |  | 0.079 | 0.618 | 0.064 | 0.701 |

Correlation analyses were performed using Pearson’s correlation on univariate and partial correlations on multivariate analysis. Multivariate analysis was adjusted for age, sex, and the presence of diabetes mellitus.

Abbreviations: *r*, correlation coefficient; ALM/Ht^2^, appendicular lean mass per height squared; FM/Ht^2^, fat mass per height squared; TMA/Ht^2^, thigh muscle area per height squared; BMD, bone mineral density; SGA, subjective global assessment; SPPB, short physical performance battery; STS5, five times sit-to-stand test; STS30, sit-to-stand for 30-second test; 6-MWT, 6-minute walk test; TUG, timed up-to-go test; hs-CRP, high-sensitivity C-reactive protein.

**Supplementary Table 7. Cox-regression analyses according to variables**

|  | **Univariate** | | **Multivariate** | |
| --- | --- | --- | --- | --- |
|  | **HR (95% CI)** | ***P*-value** | **HR (95% CI)** | ***P*-value** |
| Edema index (per increase 0.01 unit) | 2.19 (1.19–4.02) | 0.012 | 2.26 (1.15–4.43) | 0.018 |
| Gait speed (per increase 0.1 m/s) | 0.48 (0.26–0.89) | 0.019 | 0.47 (0.25–0.89) | 0.021 |
| Handgrip strength (per increase 1 kg) | 0.98 (0.87–1.10) | 0.702 | 0.97 (0.84–1.10) | 0.596 |
| SPPB (per increase 1 score) | 0.67 (0.45–1.00) | 0.050 | 0.67 (0.41–1.11) | 0.120 |
| STS5 (per increase 1 sec) | 1.08 (0.99–1.16) | 0.070 | 1.06 (0.98–1.16) | 0.155 |
| STS30 (per increase 1 time) | 0.71 (0.56–0.91) | 0.007 | 0.71 (0.55–0.91) | 0.008 |
| 6-MWT (per increase 1 meter) | 0.99 (0.99–1.00) | 0.023 | 0.99 (0.99–1.00) | 0.038 |
| TUG (per 1 sec) | 1.30 (0.95–1.77) | 0.098 | 1.26 (0.89–1.78) | 0.203 |
| ALM/Ht^2^ | 0.89 (0.39–2.02) | 0.778 | 0.73 (0.26–2.00) | 0.535 |
| TMA/Ht^2^ | 1.00 (0.96–1.03) | 0.816 | 0.99 (0.94–1.04) | 0.640 |

Multivariate analysis was adjusted for age, sex, and the presence of diabetes mellitus.

Abbreviations: HR, hazard ratio; CI, confidence interval; SPPB, short physical performance battery; STS5, five times sit-to-stand test; STS30, sit-to-stand for 30-second test; 6-MWT, 6-minute walk test; TUG, timed up-to-go test; ALM/Ht^2^, appendicular lean mass per height squared; FM/Ht^2^, fat mass per height squared; TMA/Ht^2^, thigh muscle area per height squared.
